# Are cinnamon derivatives effective and safe for diabetes?

**DOI:** 10.55730/1300-0144.5972

**Published:** 2024-12-10

**Authors:** Methiye MANCAK, Ufuk Koca ÇALIŞKAN

**Affiliations:** 1 Department of Pharmacognosy, Faculty of Pharmacy, Gazi University, Ankara, Turkiye; 2 Department of Pharmacognosy, Faculty of Pharmacy, Düzce University, Düzce, Turkiye

**Keywords:** *Cinnamomum* species, cinnamon, diabetes, high pressure liquid chromatography, enzyme inhibition

## Abstract

**Background/aim:**

Cinnamon spice is obtained by drying the tree bark of *Cinnamomum* Schaeff. species. The genus *Cinnamomum* belongs to the family Lauraceae, which comprises approximately 250 different species worldwide. The most common species on the market are *Cinnamomum verum* J. S. Presl, *C. cassia* (L.) J. Presl, *C. burmannii* (Nees & T. Nees) Blume, and *C. loureiroi* Nees. Cinnamon and its byproducts have been used for many years due to their antidiabetic effect. In the current study, the major chemical content and in vitro antidiabetic activities of different commercially available cinnamon samples were evaluated to determine whether they are safe to use for health purposes.

**Materials and methods:**

Macroscopic analyses, chromatographic analyses, and enzyme inhibition assays on diabetes-related enzymes (α-amylase, α-glucosidase, and aldose reductase) were performed on seven different samples (cinnamon sticks, tea bags, and capsules).

**Results:**

The cinnamon samples inhibited diabetes-related enzymes. The aqueous and ethanolic extracts of different cinnamon species demonstrated 7.73–333.69 mg/g of trans-cinnamaldehyde, and up to 43.73 mg/g of coumarin. Decoction and ethanolic extracts of *C. cassia*, *C. burmannii*, and *C. loureiroi* cinnamon sticks were detected to contain high levels of coumarin, which could pose a health risk, according to European Food Safety Authority data. Although antidiabetic activity was observed in the ready-made samples purchased from a herbalist, trans-cinnamaldehyde or coumarin compounds were not detected in the high-performance liquid chromatography analysis.

**Conclusion:**

The source of the cinnamon spice is crucial for the utilization of cinnamon both in food and therapeutic purposes. This research once again showed the importance of meticulous inspection of the products sold by herbalists.

## 1. Introduction

Cinnamon is a spice and herbal drug obtained by drying the bark of *Cinnamomum* (Lauraceae) Schaeff. species. About 250 different species of *Cinnamomum* have been identified all over the world [1]. The import of cinnamon to Europe began when the Portuguese discovered cinnamon trees growing in Sri Lanka (Ceylon) in the early 16th century [2]. Today, cinnamon trees are produced in China, Indonesia, Vietnam, India, Laos, Seychelles, and Madagascar, and especially in Sri Lanka.

Cinnamon is derived from the Greek word ‘kinnamomon’, which means sweet tree [3]. Although cinnamon is used as the common name for many different *Cinnamomum* species, true cinnamon is the dried inner bark of *Cinnamomum verum* J. S. Presl (syn. *C. zeylanicum*) (Lauraceae) [4]. It is also known as Sri Lanka cinnamon or Ceylon cinnamon. Other types of cinnamon commonly traded are *C. cassia* (L.) J. Presl (Chinese cinnamon), *C. burmannii* (Nees & T. Nees) Blume (Indonesian cinnamon), and *C. loureiroi* Nees (Vietnamese cinnamon).

Ceylon cinnamon is native to Sri Lanka, and is obtained from the inner bark of *C. verum*, which is peeled from the cork layer before drying. Thin barks are intertwined, soft, fluffy, light colored, and dried in cylindrical form. The bark of other cinnamon species is hard, dark, hollow, and in the form of a single-layered roll [5]. Although Ceylon cinnamon differs from cassia-type cinnamon (Chinese, Indonesian, and Vietnamese cinnamon) in terms of color, smell, taste, and appearance, the most important difference is in the coumarin, which is found in trace amounts in Ceylon cinnamon, while cassia-type cinnamon contains relatively higher levels [6]. The bark of Ceylon cinnamon, namely *Cinnamomi cortex*, its tincture, namely *Cinnamomi corticis tincture*, and the essential oil obtained by steam distillation from the leaves, namely *Cinnamomi zeylanici folii aetheroleum*, are registered in the European Pharmacopoeia (EP) [7].

*C. burmannii*, also known as Koerintji cinnamon or Java cinnamon, originated mainly from Sumatra, and is an endemic species in Indonesia [8]. Indonesian cinnamon has a high cinnamaldehyde content. Nonetheless, it is not a type of cinnamon registered in the EP.

*C. cassia* (=*C. aromaticum*) is cultivated in East Asian countries such as Burma (Myanmar), India, Malaysia, Indonesia, Laos, Thailand, Taiwan, Vietnam, and Southern China [9]. It is a type of cinnamon that is widely used all over the world. Only the essential oil, known as *Cinnamomi cassiae aetheroleum*, obtained by steam distillation from its young branches and leaves, is registered in the EP [7].

*C. loureiroi*, also called Saigon or Vietnamese cinnamon, is native to Southeast Asia, and has a stronger aroma, and higher cinnamaldehyde and essential oil content than the other types of cinnamon [10]. Vietnamese cinnamon is not registered in the EP.

Diabetes is a condition characterized by chronically high blood sugar due to insufficient insulin secretion and/or the body’s resistance to insulin [11]. According to statistical studies, the number of adults with diabetes worldwide reached 537 million by 2021, and it is estimated that this number will increase to 643 million by 2030 and 783 million by 2045 [12]. *Cinnamomum* bark is used as folk medicine to regulate blood sugar, and its effect on diabetes has been demonstrated by scientific in vitro, in vivo, and clinical research [13–16]. Since a current treatment method for diabetes is the inhibition of enzymes involved in the hydrolysis of carbohydrates in the digestive tract, in vitro studies are being conducted on the inhibition of diabetic enzymes (i.e. α-amylase, α-glucosidase, aldose reductase) [14,17,18]. Inhibitors of these enzymes restrict the digestion of dietary carbohydrates and thereby inhibit the absorption of simple sugars, which causes low postprandial glucose levels in the blood. Increased utilization of glucose by the aldose reductase enzyme plays a serious role in the occurrence of diabetic complications [19]. Inhibition of the aldose reductase enzyme has been shown to prevent diabetes complications, especially cataracts and retinopathy [20].

In the current study, the antidiabetic activities of the extracts of various *Cinnamomum* species and cinnamon-containing products obtained from the market were analyzed by in vitro α-amylase and α-glucosidase as well as aldose reductase enzyme inhibition assays. Moreover, the safe usability of the samples was assessed due to their high coumarin (1-benzopyran-2-one) content, which has been shown to be hepatotoxic, genotoxic, and carcinogenic, in animal studies [21,22]. Since coumarin is present in the composition of foods consumed in daily life, The European Food Safety Authority (EFSA) and The German Federal Institute for Risk Assessment (BfR) set the tolerable daily intake (TDI) for coumarin at 0.1 mg/kg of body weight [23,24]. Based on this, the coumarin content of the cinnamon samples was determined herein. At the same time, the amount of trans-cinnamaldehyde, the major component of the samples, was also investigated.

## 2. Materials and methods

### 2.1. Plant samples

A total of seven samples were studied. Four cinnamon sticks were purchased from herbalists and markets. One tea bag containing cinnamon was purchased from the herbalist, and one of the two capsules containing cinnamon spice was purchased from the herbalist on their recommendation. The other capsule was obtained from a pharmacy.

### 2.2. Macroscopic analysis

Macroscopic analyses using the *Cinnamomi cortex* monograph in EP 8.0 were performed on the cinnamon stick samples. The general appearance, size, and color of the samples were determined by macroscopic analysis.

### 2.3. Extract preparation

Ten of each cinnamon stick species were weighed individually. Then, the average weight of a cinnamon stick was calculated for each species. Next, ethanol and aqueous extracts of the cinnamon sticks were prepared. For the ethanol extract, 500 mL of ethanol was added to 50 g of the plant samples and macerated at room temperature. Aqueous extracts were prepared with two different preparation techniques, infusion and decoction. For the infusion, boiled distilled water was added to 50 g of the samples and infused for 15 min. For the decoction, distilled room temperature water was added to the samples, which were then boiled for 10 min. The procedures were repeated three times. The average weight of one cinnamon stick and the yield of the extracts are presented in Table 1.

For a sample of cinnamon-containing tea purchased from herbalists, a filtering bag was placed in a cup (120 mL) of hot water. The filtrate obtained was concentrated with a lyophilizer and used in the analysis.

Capsules were dissolved in methanol separately and stock solutions were prepared. The solutions were used in the analyses by making appropriate dilutions. Although the capsules obtained from the herbalist were stated to contain 300 mg, the content was found to be in the range of 280–285 mg, and the calculations were made considering this amount weighed.

### 2.4. Chromatographic analysis

#### 2.4.1. Thin-layer chromatography (TLC)

TLC analyses were performed according to the conditions specified in the *Cinnamomi cortex* monograph in EP 8.0. Cinnamic aldehyde, eugenol, and coumarin standards were dissolved with toluene, and a TLC silica gel GF254 plate was applied, and examined at 254 and 366 nm.

#### 2.4.2. High-performance liquid chromatography (HPLC)

The Agilent model was performed using a device and a C18 (150 × 4.6 mm, 5 μm) column. The amounts of trans-cinnamaldehyde and coumarin in the cinnamon samples were determined. The analysis conditions were designed by modifying the studies in the literature. As such, the mobile phase used water containing formic acid (solvent A) and acetonitrile (solvent B), at a flow rate of 1 mL/min. The gradient flows were as follows: 1 min, 5% B; 31 min, 28% B; 36 min, 30% B; 45 min, 70% B; 49 min, 90% B; 50 min, 5% B, and hold at 5% B for 5 min. The injection volume was 10 μL and the wavelength was 320 nm.

### 2.5. In vitro enzyme activity assays

The inhibition effects of the cinnamon samples at different concentrations on the following enzymes were investigated: α-amylase, α-glucosidase, and aldose reductase.

#### 2.5.1. α-Amylase enzyme inhibition assay

The α-amylase enzyme inhibition assay was performed by modifying the in vitro method used by Eruygur et al. [25]. First, 50 μL of amylase enzyme (0.06 M NaCl, in 0.02 M, pH 6.9 phosphate buffer) was added to the plant extracts and incubated at 37 °C for 10 min. Afterward, 50 μL of the starch solution was added and incubated for another 10 min. After incubation, 25 μL of HCl and 100 μL of lugol solution were added. The absorbance was measured spectrophotometrically at 540 nm. Acarbose was used as a reference substance.

#### 2.5.2. α-Glucosidase enzyme inhibition assay

The α-glucosidase enzyme inhibition assay was performed by modifying the in vitro method used by Eruygur et al [25]. First, 50 μL of glucosidase enzyme was added to the plant extracts and incubated at 37 °C for 5 min. Then, 50 μL of p-nitrophenyl-α-D-glucopyranoside was added and incubated for a second time at 37 °C for 30 min. At the end of incubation, absorbances were read at 405 nm. Acarbose was used as a reference substance.

#### 2.5.3. Aldose reductase enzyme inhibition assay

The aldose reductase enzyme inhibition effect of the samples was determined by modifying the in vitro method of Hayman and Knoshita [26]. Homogenizer obtained from rabbit lenses was used as the source of the aldose reductase enzyme. The rabbit lenses were homogenized in 100 mM of phosphate buffer (pH 6.9) and a 10% homogenizer was prepared. The plant extracts, 25 μL of NADPH, and 25 μL of homogenate were added to 100 μL of potassium buffer. The mixture was incubated at 37 °C for 5 min. At the end of incubation, DL-glyceraldehyde was added, and the NADPH change was observed at 340 nm at 37 °C for 15 min. Quercetin was used as a positive control.

## 3. Results

### 3.1. Macroscopic analysis

**Figure 1:**
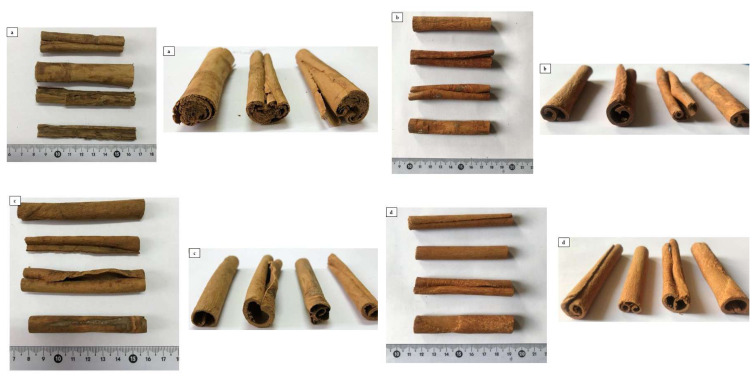
Images of the cinnamon stick samples: a) *C. verum*, b) *C. burmannii*, c) *C. cassia*, and d) *C. loureiroi*.

As shown in Figure 1a, the *C. verum* cinnamon stick samples can be easily distinguished from others in terms of their morphologically intertwined layers, color, odor, and fragility. According to the macroscopic analyses, the *C. verum* bark samples were 1–2.5 cm thick, 8–9 cm long, brown, had a cigar-like appearance, an aromatic smell, and tasted sweet. Both sides of the shell were curved inward, and consisted of intertwined, thin, and fragile layers. As shown in Figure 1b, the *C. burmannii*, Indonesian cinnamon bark samples were irregularly curved in lateral view and usually hollow, very hard, reddish-brown, with fungal layers detected on the outer surface, 1–1.5 cm thick, and 8–8.5 cm long. As shown in Figure 1c, the *C. cassia* bark samples were dark reddish-brown 1–1.5 cm thick, and 8–9 cm long, with fungal layers observed on the outer surface, which were hollow, tubular shaped, irregular, and usually curled in the side view, with a spicy smell and a spicy-bitter taste. On the other hand, as shown in Figure 1d, the *C. loureiroi* bark samples were light brown, tubular, hollow, and generally composed of a single layer, 1–1.5 cm thick, and 8–9 cm long. The findings of the analyses of these cinnamon stick samples were consistent with the studies in the literature [27,28].

### 3.2. Chromatographic analysis

#### 3.2.1. TLC

**Figure 2:**
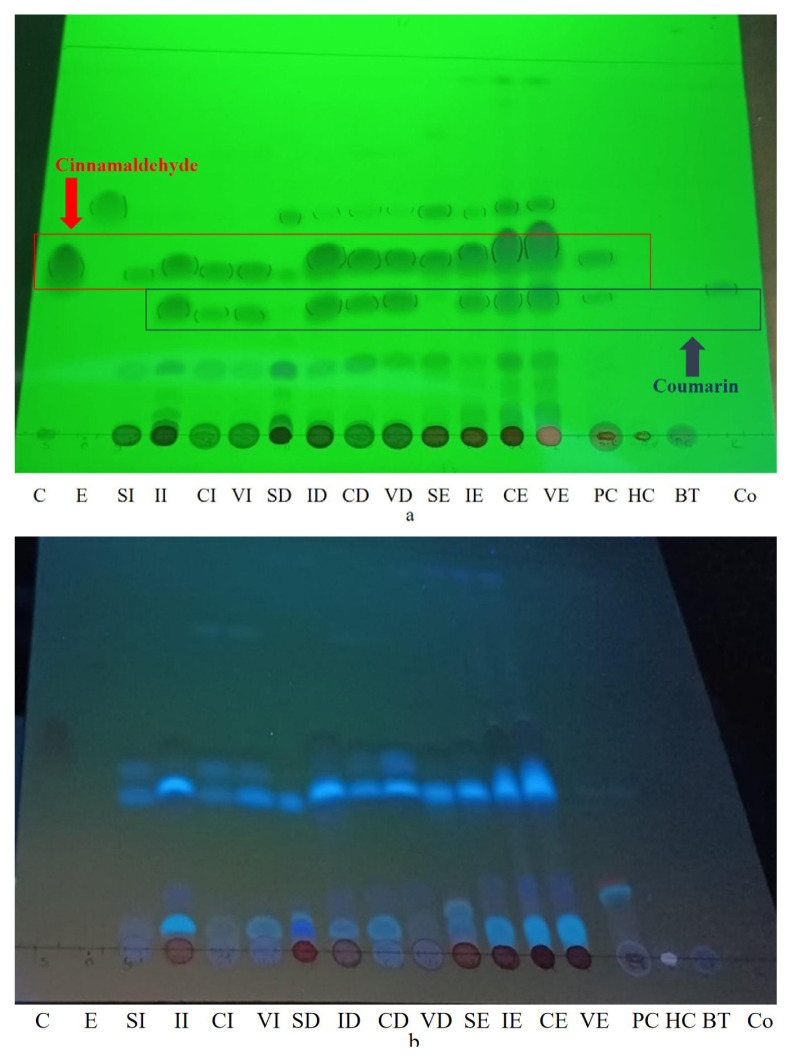
TLC plate UV254 (a) nm and UV366 (b) images of the cinnamon samples. C: Cinnamaldehyde, E: Eugenol, Co: Coumarin, SI: Sri Lanka-infusion, SD: Sri Lanka-decoction, SE: Sri Lanka-ethanol, II: Indonesia-infusion, ID: Indonesia decoction, IE: Indonesia-ethanol, CI: Chinese-infusion, CD: Chinese decoction, CE: Chinese ethanol, VI: Vietnamese-infusion, VD: Vietnamese-decoction, VE: Vietnamese-ethanol, PC: pharmacy capsule, HC: herbalist capsule, and BT: bag tea.

The analysis of the silica gel plate at 254 nm, as shown in Figure 2a, showed a cinnamaldehyde zone in the middle of the plate, an eugenol zone just above the cinnamaldehyde, and a coumarin zone below the cinnamaldehyde. As shown in Figure 2b, the o-methoxy-cinnamaldehyde stain appeared as a light blue, fluorescent stain just below the cinnamaldehyde zone at 366 nm. According to the TLC plate, a cinnamaldehyde zone was observed in all the cinnamon stick extracts and the food supplement sample obtained from the pharmacy. No coumarin was detected in any extracts of the *C. verum*, while a coumarin zone was detected in all the other cinnamon stick extracts and the dietary supplement sample. Eugenol zones were determined in the decoction and ethanol extracts of the cinnamon sticks. None of the cinnamaldehyde, eugenol, or coumarin stains were found in the capsule or tea bag samples obtained from the herbalist. An O-Methoxycinnamaldehyde zone was detected in the cinnamon stick extracts.

#### 3.2.2. HPLC

**Figure 3:**
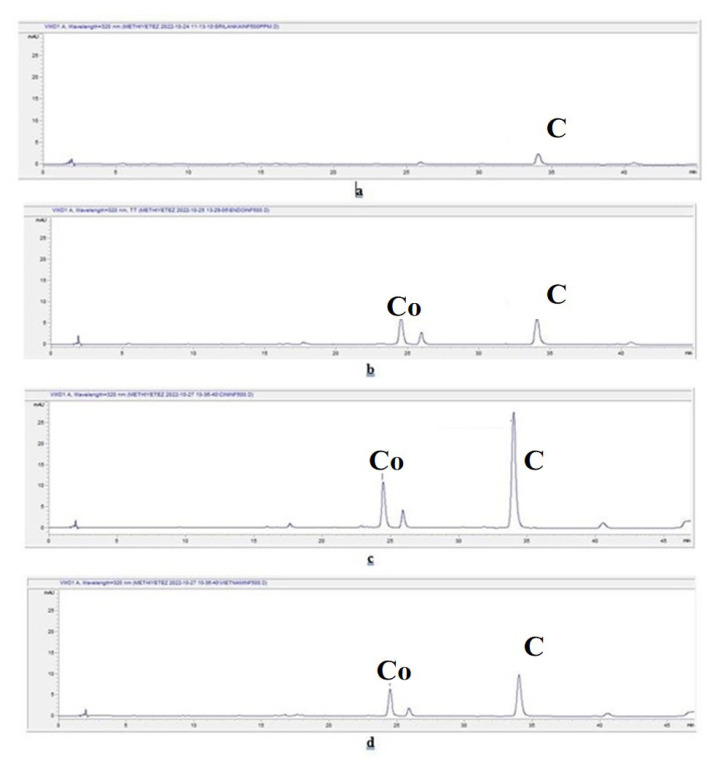
Chromatograms of the aqueous extracts prepared by infusion of cinnamon stick samples. C: Cinnamaldehyde. Co: Coumarin. **a.** *C. verum* infusion extract chromatogram, **b.** *C. burmannii* infusion extract chromatogram, **c.** *C. cassia* infusion extract chromatogram, and **d.** *C. loureiroi* infusion extract chromatogram.

**Figure 4:**
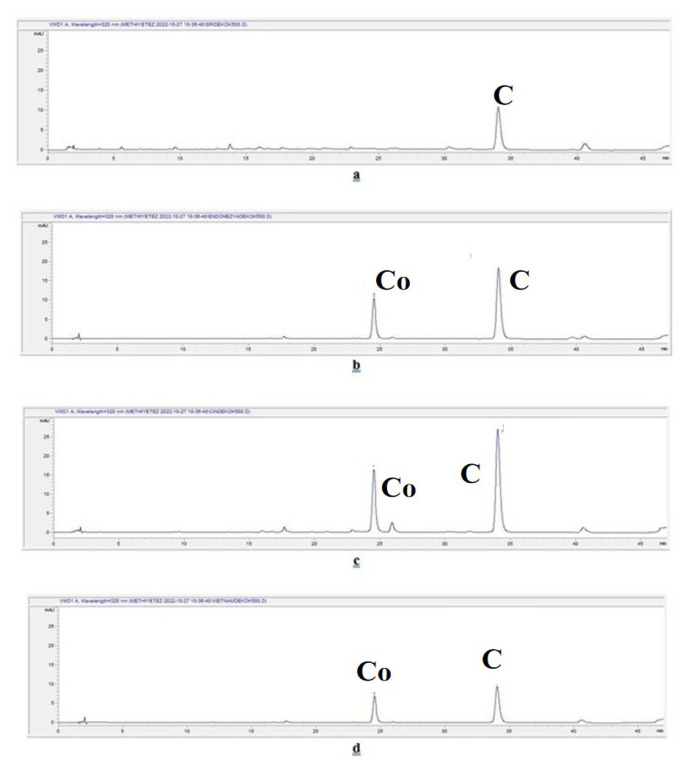
Chromatograms of the aqueous extracts prepared by decoction of the cinnamon stick samples. C: Cinnamaldehyde. Co: Coumarin. **a.** *C. verum* decoction extract chromatogram, **b.** *C. burmannii* decoction extract chromatogram, **c.** *C. cassia* decoction extract chromatogram, and **d.** *C. loureiroi* decoction extract chromatogram.

**Figure 5:**
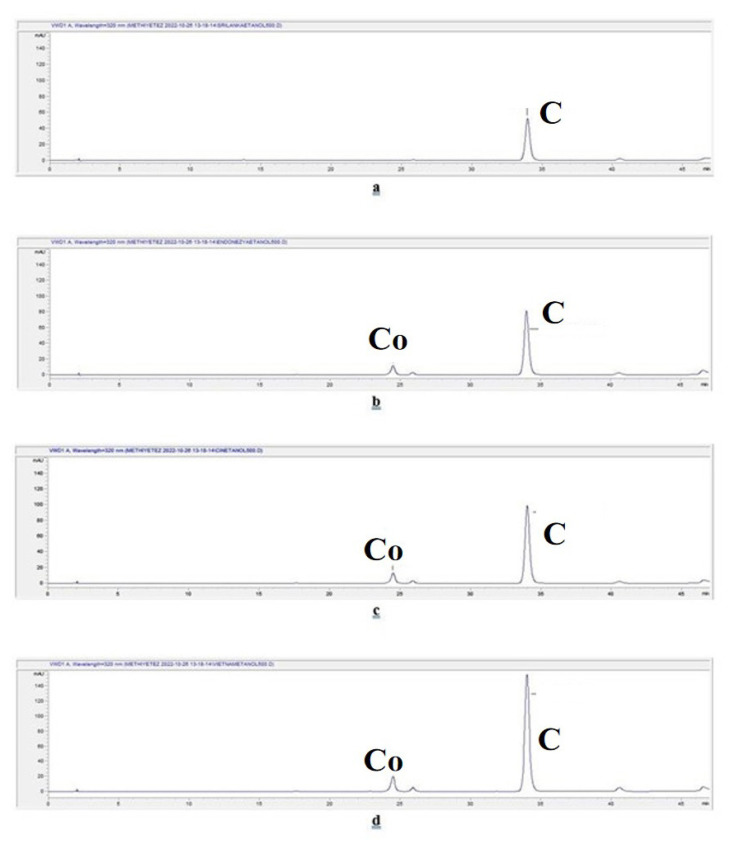
Chromatograms of the ethanol extracts of the cinnamon stick samples. C: Cinnamaldehyde. Co: Coumarin. **a.** *C. verum* ethanol extract chromatogram, **b.** *C. burmannii* ethanol extract chromatogram, **c.** *C. cassia* ethanol extract chromatogram, and **d.** *C. loureiroi* ethanol extract chromatogram.

**Figure 6:**
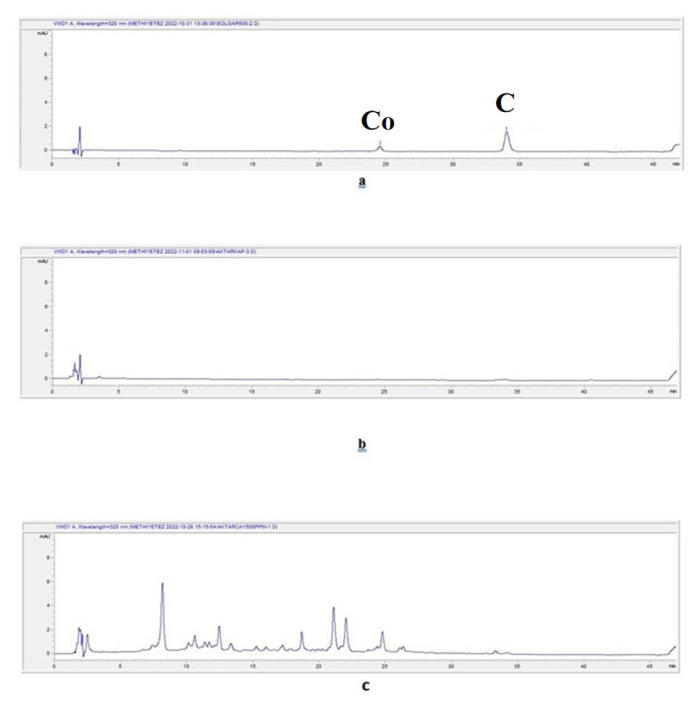
Chromatograms of the ready-made cinnamon samples. C: Cinnamaldehyde. Co: Coumarin. **a.** Food supplement cinnamon capsules available from the pharmacy, **b.** Cinnamon capsules obtained from the herbalist, and **c.** Tea bag containing cinnamon from herbalist.

The chromatograms of the aqueous extracts prepared by the infusion method for *C. verum*, *C. burmannii*, *C. cassia*, and *C. loureiroi* are given in Figures 3a–3d, respectively. The chromatograms of the aqueous extracts prepared by the decoction method for *C. verum*, *C. burmannii*, *C. cassia*, and *C. loureiroi* are given in Figures 4a–4d, respectively. The chromatograms of the extracts prepared using ethanol for *C. verum*, *C. burmannii*, *C. cassia*, and *C. loureiroi* are given in Figures 5a–5d, respectively. The chromatograms of the cinnamon capsules from the pharmacy, cinnamon capsules obtained from the herbalist, and tea bag containing cinnamon from the herbalist are shown in Figures 6a–6c, respectively.

The equation for trans-cinnamaldehyde was y = 25.22x – 43.424 (R^2^: 0.9994; limit of detection (LOD): 0.2799; limit of quantification (LOQ): 0.8480). The equation for coumarin was y = 23.161x + 14.758 (R^2^: 0.9994; LOD: 0.0747; LOQ: 0.2263). The amounts of trans-cinnamaldehyde and coumarin detected in the cinnamon samples are given in Table 2. The amount of trans-cinnamaldehyde in a cinnamon stick was calculated from the yields of the extracts and expressed as the amount per unit. The presence of trans-cinnamaldehyde was observed by chromatographical methods both by TLC and by HPLC analyses in all the extracts of the cinnamon stick samples and the cinnamon capsule obtained from the pharmacy, excluding the cinnamon capsule and tea bag obtained from the herbalist. Although the maximum amount of trans-cinnamaldehyde was reported in the essential oil of cinnamon bark in the EP, no such value was specified directly in cinnamon bark. Analysis revealed that the ethanol extracts of the cinnamon contained more trans-cinnamaldehyde than the aqueous extracts for all the cinnamon stick samples. Among the aqueous extracts, the amount of trans-cinnamaldehyde in the extracts prepared by the decoction method was higher. The highest trans-cinnamaldehyde content among the cinnamon sticks was determined in the *C. loureiroi* ethanol extract. When the infusion and decoction extracts of the samples were compared, *C. cassia* had the highest trans-cinnamaldehyde content in both. It was found that one capsule of the dietary supplement samples obtained from the pharmacy contained 3.9519 ± 1.1153 mg of trans-cinnamaldehyde. Trans-cinnamaldehyde was not detected in the chromatograms of the capsule and tea bag samples obtained from the herbalist. The literature revealed that *C. cassia* cinnamon contains much higher amounts of trans-cinnamaldehyde than *C. verum* cinnamon [6]. In line with this, the trans-cinnamaldehyde content of *C. cassia* was found to be more than twice that of *C. verum* in the current study. He et al. [29] reported that methanol extracts from the bark of *C. cassia* contained high amounts of cinnamaldehyde (the highest was 93.83 mg, 13.01–56.93 mg/g), while *C. burmannii* bark contained low amounts (<2 mg/g). They cited the Chinese Pharmacopoeia, which states a good quality cinnamon bark should contain more than 10 mg/g of cinnamaldehyde and considered the bark of *C. cassia* as good quality and bark the *C. burmannii* species as ‘fake’ cinnamon bark. In the present study, extracts from cinnamon bark were prepared with ethanol that had polarity similar to methanol. Within the scope of the study, it was determined that the amount of cinnamaldehyde in the ethanol extracts of not only *C. cassia* but also *C. loureiroi* was higher than 10 mg/g. Moreover, the trans-cinnamaldehyde content of *C. burmannii* was close to that of these species. On the other hand, the literature revealed that the amount of trans-cinnamaldehyde in *C. cassia* species is unacceptably high [6]. In the study of Lungarini et al. [23], toxicity evaluations of cinnamaldehyde were conducted, and they attempted to determine acceptable intake doses in various safety evaluations, but no safety issues regarding its consumption were found. On the other hand, it has been suggested that it may pose a risk to human health due to toxicity in experimental studies [30,31].

According to the results, the cinnamon sticks showed similar results macroscopically, the differences in the coumarin content were decisive in the distinction. No coumarin was detected in any extract of *C. verum*. As stated in the literature, *C. verum* contains much lower amounts of coumarin than other cinnamon types [6,32]. Although the LOD and LOQ values of coumarin (0.0747 and 0.2263, respectively) were quite low, no coumarin peak was observed in the chromatogram of *C. verum* and the amount of it could not be determined. This showed that *C. verum* contains trace amounts of coumarin.

In the other cinnamon stick extracts, very high amounts of coumarin were detected, mostly in the ethanol extracts. The highest amount of coumarin was determined in the *C. loureiroi* ethanol extract. Although a small peak of coumarin was observed on the HPLC chromatogram of the capsules obtained from the pharmacy, it could not be calculated because the amount was below the LOD value. No coumarin peak was determined in the capsules and tea bags supplied by the herbalists and the amount of coumarin could not be determined.

The amount of coumarin for cinnamon bark is also not specified in the EP. The approximate amount of coumarin in one cinnamon stick was also calculated since coumarin is a risky component with a defined daily intake dose. Calculations were based on the calculated average weights of cinnamon sticks and the yield of the extract, and the results are presented in Table 2. When evaluated in terms of maintaining health, the TDI limit for coumarin was set by the EFSA as 0.1 mg/kg, and this limit is 7 mg for an adult weighing 70 kg. In the current study, based on the extract yields, the approximate amount of coumarin ingested by individuals when they use one stick of cinnamon extracted in different ways was calculated. As a result, *C. burmannii*, *C. cassia*, and *C. loureiroi* species were detected to contain coumarin below the daily intake limit when consumed by the infusion of a cinnamon stick. *C. cassia* and *C. loureiroi* ethanol extracts were determined to contain coumarin well above the daily intake limit. Consumption of aqueous extracts of cinnamon sticks of the species *C. burmannii* and *C. cassia* prepared by decoction, that is by boiling, exceeded the daily intake limit for coumarin. In fact, the percent coumarin contents of *C. burmannii* ethanol extract and *C. loureiroi* decoction extract were high. However, due to the low yield of these extracts, the amount of coumarin taken in a stick of cinnamon consumption was below the daily intake limit. These data revealed the importance of the solvent from which the extract was prepared and the preparation technique. Although the *C. burmannii*, *C. cassia*, and *C. loureiroi* cinnamon stick samples were found to be safe to use via brewing with hot water without boiling, the low levels of coumarin found even in the ethanol extract of *C. verum* indicated that this species was safer to use than the cassia type cinnamon. Similarly, the coumarin content of the capsules obtained from the pharmacy was not detected because it was lower than the LOD value, which created confidence in the products obtained from the pharmacy.

### 3.3. In vitro enzyme activity assays

The results of the in vitro enzyme inhibition analysis of the cinnamon samples are presented in Table 3. The ethanol extracts of the cinnamon sticks showed higher enzyme inhibition than aqueous extracts. *C. cassia* showed the highest inhibition on the α-amylase and aldose reductase enzymes, while *C. burmannii* showed the strongest activity on the α-glucosidase enzyme.

Beejmohun et al. [15], in their study with Ceylon cinnamon, determined its inhibitory effect on the α-amylase enzyme, which was lower than acarbose (half-maximal inhibitory concentration (IC_50_): 25 and 18 μg/mL, respectively). They showed that the preparation of cinnamon as a hydroalcoholic extract increased the inhibition of the α-amylase enzyme compared to the extracts prepared with only water. In the current study, the ethanol extracts of all the cinnamon species analyzed had stronger α-amylase inhibition than the aqueous extracts. The cinnamon species showed a weaker effect than acarbose but clearly exhibited significant inhibition on α-amylase. Adisakwattana et al. [33] prepared extracts of four cinnamon bark species with distilled water at 90 °C and compared their effects on the α-amylase enzyme. They determined that all the cinnamon extracts inhibited the α-amylase enzyme significantly, but the effect was lower than the reference substance acarbose. Ceylon cinnamon showed the strongest effect on the α-amylase enzyme and Vietnamese cinnamon showed the weakest. Hayward et al. [17] investigated the α-amylase inhibition activity of cinnamon extracts prepared using ethanol-water. They found that *C. cassia* extract (IC_50_: 5.3 μg/mL) showed significantly higher efficacy that was similar to acarbose (IC_50_: 4.2 μg/mL) compared to other types of cinnamon. In the current study, as in theirs, Chinese cinnamon was the most effective on α-amylase. However, the species with the weakest effect was Indonesian cinnamon, contrary to the studies mentioned above. This difference may have been due to differences in the solvents in which the extracts were prepared, as well as differences in the experimental conditions (temperature, substrate type, enzyme type, buffer conditions, incubation temperature, and duration). α-Amylase enzyme inhibition by the capsule obtained from the pharmacy stated to contain *C. burmannii* showed results similar to the ethanol extract prepared from the *C. burmannii* cinnamon stick. The cinnamon capsules from the pharmacy showed higher inhibition than those from herbalists. The α-amylase inhibition levels of the capsules were higher than the aqueous extracts of the cinnamon sticks prepared by the infusion and decoction methods. The tea bag obtained from the herbalist, which was referred to as cinnamon tea and contained a mixture of many herbs, showed results similar to the aqueous extracts.

*C. burmannii* showed the highest activity on the α-glucosidase enzyme and *C. loureiroi* species showed the lowest. *C. verum* had high efficacy, similar to that of *C. burmannii*. Although the α-glucosidase enzyme inhibition activity of the cinnamon stick extracts was lower than that of the reference substance acarbose, it was comparable. Hayward et al. [17] evaluated the α-glucosidase enzyme inhibition of cinnamon species in their study, and found that the cinnamon species had stronger activity on α-glucosidase when compared to α-amylase. As in their study, the cinnamon species herein showed higher activities on α-glucosidase at much lower doses. Moreover, in their study, Chinese cinnamon and Indonesian cinnamon had the highest activity, while in the present study, Indonesian and Sri Lankan cinnamon had the strongest activity [17]. Similar to the current study, Sri Lankan cinnamon exhibited the highest activity on α-glucosidase in the study by Adisakwattana et al. [33]. Cinnamon capsules from the pharmacy were found to have higher inhibition than the capsules and tea bags from the herbalists.

All the cinnamon species extracts had lower activity on the aldose reductase enzyme than the quercetin used as a reference substance. Extracts of all the species prepared using ethanol showed higher activity than the aqueous extracts. When the literature was examined, limited studies were found on the aldose reductase enzyme inhibition effect of cinnamon. A study investigating the effect of *C. cassia* methanol extract and its fractions on aldose reductase was conducted by Lee [18]. They found that the methanol extract of the plant, like the reference substance quercetin, provided 100% inhibition on the aldose reductase enzyme at 0.1 mg/mL. However, the amount of inhibition of the extract at the doses used was not included in their study. According to the study data, the IC_50_ value of quercetin was 0.0005 mg/mL, and the IC_50_ values of the components were included, but the IC_50_ value of the methanol extract was not reported. Since no other study examining the effect of cinnamon samples on the aldose reductase enzyme could be found in the literature, a comparison with the literature data could not be made. Therefore, to our knowledge, this is the first study to compare the effect of different species of cinnamon on the aldose reductase enzyme. This study found that the cinnamon samples had an effect on the aldose reductase enzyme at a dose of 50 μg/mL and this effect was lower than quercetin.

## 4. Conclusion

In an overview of the results of the study, the cinnamon samples had antidiabetic activities in vitro. The higher activity of the ethanol extracts in the enzyme assays showed that ethanol is an ideal solvent for all three enzymes and is effective in revealing the components in the plant content that cause enzyme inhibition. On the other hand, it was understood that the teas prepared by people at home using water were not ineffective.

The most important milestone of this study was the determination that not everything with positive in vitro activity is safe. Although capsules or tea bags from herbalists are effective on diabetes enzymes, there were issues with the ingredients of these samples. Trans-cinnamaldehyde, coumarin, and o-methoxy cinnamaldehyde were not detected in either the TLC plate or the HPLC chromatogram for the cinnamon capsule sample obtained from the herbalist. This made us think that the effectiveness of this sample was not due to cinnamon. Moreover, this product claimed to contain 275 mg of cinnamon extract and 25 mg of termite seed powder. However, after weighing, it was determined that the total content was between 280 and 285 mg. Moreover, although the components of cinnamon, the main ingredient, could not be identified, it showed inhibitory activity on the diabetic enzymes, suggesting that different substances were added to its content, causing safety concerns. Even for herbalists in different provinces, this is one of the products most recommended by them for diabetes. Considering all of these, concerns are increasing, and this shows once again the importance of immediate and rigorous inspection of the products sold by herbalists. These data question the reliability of ready-made preparations obtained from outside the pharmacy.

Although the preparations obtained from the pharmacy were dietary supplements and not approved by the Ministry of Health, they showed inhibitory activity on the diabetes enzymes and no issues were found with their content. This made us think that even if the products sold in pharmacies are not approved by the Ministry of Health, they are selected more carefully than the products sold outside the pharmacy, and the those offered by pharmacists to patients/consumers may be more reliable.

The results of the analyses conducted with different species of cinnamon plants from different countries revealed that the type of cinnamon to be used for both food and therapeutic purposes is vital. Decoctions and ethanol extracts prepared from cinnamon species other than Sri Lankan cinnamon were found to contain a coumarin content high enough to pose a health risk according to EFSA. Based on these data, it is not possible to remove cassia-type cinnamon from the market from a public health point of view, but it is crucial that consumers are made aware of its use according to the purpose and method of use. Considering that Sri Lankan cinnamon can be easily distinguished from other cinnamon, consumers should be informed about the distinction between cinnamon types and directed to consume Sri Lankan cinnamon. In cases where individuals obtain cinnamon in powder form or cannot distinguish it, cinnamon should be consumed without boiling or other processing, considering the dose.

## Figures and Tables

**Table 1:** Average weights of the cinnamon stick samples and percent yields of the extracts.

Samples (cinnamon stick)		% extract yield	Average weight of a cinnamon stick
*C. verum*	Infusion	5.236	5.01 ± 1.1007 g
	Decoction	7.860	
	Ethanol extract	5.182	
*C. burmannii*	Infusion	3.384	4.662 ± 2.3266
	Decoction	11.710	
	Ethanol extract	6.122	
*C. cassia*	Infusion	7.418	3.602 ± 1.3908
	Decoction	11.151	
	Ethanol extract	7.596	
*C. loureiroi*	Infusion	7.586	3.642 ± 1.2454
	Decoction	5.514	
	Ethanol extract	6.676	

**Table 2:** Results of the trans-cinnamaldehyde and coumarin quantification of the cinnamon samples.

Sample name		Trans-cinnamaldehyde in 100 ppm extract	Trans-cinnamaldehyde in 1 gram extract (mg/g extract)	* Quantity per unit (mg)	** Trans-cinnamaldehyde in 1 g of the form (mg/g)	% coumarin	Coumarin in 1 gram extract (mg/g extract)	* Quantity per unit (mg)
***C. verum***	Infusion	0.77 ± 0.01	7.73 ± 0.17	2.02 ± 0.04	0.40 ± 0.01	-	-	-
	Decoction	2.20 ± 0.14	22.01 ± 1.43	8.66 ± 0.56	1.73 ± 0.11	-	-	-
	Ethanol extract	10.67 ± 0.64	106.73 ± 6.46	27.70 ± 1.67	5.53 ± 0.33	-	-	-
***C. burmannii***	Infusion	1.75 ± 0.46	17.51 ± 4.64	2.76 ± 0.73	0.59 ± 0.15	0.94 ± 0.04	9.44 ± 0.46	1.49 ± 0.07
	Decoction	4.11 ± 0.65	41.14 ± 6.50	22.46 ± 3.54	4.81 ± 0.76	1.42 ± 0.23	14.26 ± 2.32	7.78 ± 1.26
	Ethanol extract	16.23 ± 0.73	162.32 ± 7.37	46.32 ± 2.10	9.93 ± 0.45	2.03 ± 0.05	20.308 ± 0.58	5.79 ± 0.16
***C.cassia***	Infusion	5.34 ± 0.36	53.40 ± 3.7	14.26 ± 0.98	3.96 ± 0.27	1.51 ± 0.11	15.177 ± 1.13	4.05 ± 0.30
	Decoction	5.49 ± 0.41	54.95 ± 4.15	22.07 ± 1.67	6.12 ± 0.46	2.49 ± 0.09	24.91 ± 0.97	10.01 ± 0.39
	Ethanol extract	22.97 ± 5.83	229.71 ± 58.32	62.85 ± 15.95	17.44 ± 4.43	2.83 ± 0.71	28.34 ± 7.16	7.75 ± 1.96
***C. loureiroi***	Infusion	2.07 ± 0.06	20.75 ± 0.63	5.73 ± 0.17	1.57 ± 0.03	0.89 ± 0.01	8.94 ± 0.05	2.47 ± 0.01
	Decoction	2.39 ± 0.48	23.95 ± 4.89	4.81 ± 0.98	1.32 ± 0.27	0.97 ± 0.04	9.74 ± 0.42	1.95 ± 0.08
	Ethanol extract	33.36 ± 5.46	333.69 ± 54.64	81.13 ± 13.28	22.27 ± 3.65	4.37 ± 1.09	43.73 ± 10.91	10.63 ± 2.65
**Capsule from pharmacy**		0.79 ± 0.22	7.90 ± 2.23	3.95 ± 1.11	0.01 ± 0.002	-	-	-
**Capsule from herbalist**		-	-	-	-	-	-	-
**Bag tea**		-	-	-	-	-	-	-

* Quantity per unit: one unit of the presented form of sample. One cinnamon stick, one capsule, or one tea bag,

** the mg amount of trans-cinnamaldehyde in 1 g of the form: the amount in mg of trans-cinnamaldehyde in 1 g cinnamon stick, 1 g of capsule, or 1 g of tea bag.

**Table 3:** Results of the in vitro enzyme inhibition analysis of the cinnamon samples.

		α-amylase enzyme inhibition (%inhibition±standard deviation)				α-glucosidase enzyme inhibition (%inhibition±standard deviation)			Aldose reductase enzyme inhibition (%inhibition± standard deviation)	
Sample name		6.75 μg/mL	12.5 μg/mL	25 μg/mL	50 μg/mL	2.5 μg/mL	5 μg/mL	10 μg/mL	25 μg/mL	50 μg/mL
*C. verum*	Infusion	50.07± 0.53	57.71 ± 1.16	66.52 ± 0.28	83.73 ± 1.64	37.89 ± 0.29	48.36 ± 1.18	81.12 ± 0.11	12.97 ± 1.12	47.10± 1.69
	Decoction	55.38± 0.12	59.52 ± 1.65	72.95 ± 0.40	99.99 ± 0.01	43.69 ± 2.05	85.17 ± 1.56	95.26 ± 0.11	20.10 ± 3.27	46.95± 0.13
	Ethanol extract	64.49± 1.13	71.72 ± 1.10	98.51 ± 1.37	99.99 ± 0.01	81.70 ± 0.21	92.38 ± 2.04	97.95 ± 0.02	40.26 ± 0.11	69.34 ± 0.12
*C. cassia*	Infusion	44.08± 3.57	89.94 ± 1.46	99.99 ± 0.01	99.99 ± 0.1	57.25 ± 3.25	66.99 ± 2.96	80.65 ± 0.07	36.91 ± 0.12	51.01± 1.14
	Decoction	67.92± 0.96	84.03 ± 1.19	97.52 ± 4.54	99.99 ± 0.01	72.21 ± 0.13	79.56 ± 1.27	97.59 ± 0.16	48.02 ± 1.15	62.27± 2.44
	Ethanol extract	80.10± 0.59	91.23 ± 2.84	99.99 ± 0.01	99.99 ± 0.01	65.08 ± 0.10	88.81 ± 0.09	98.02 ± 0.14	39.98 ± 1.15	69.43± 1.24
*C. burmannii*	Infusion	12.64± 2.19	17.85 ± 1.21	38.56 ± 5.14	68.26 ± 0.63	58.22±0.54	70.42 ± 0.06	86.56 ± 2.02	22.69 ± 0.01	43.87± 0.13
	Decoction	25.57± 0.33	29.02 ± 0.23	35.31 ± 1.76	44.79 ± 2.88	60.93 ± 4.16	83.53 ± 0.59	96.91 ± 0.03	27.34 ± 0.20	45.40± 0.16
	Ethanol extract	30.24± 2.81	54.17 ± 5.60	82.28 ± 3.89	89.93 ± 3.49	90.98 ± 1.61	95.2 ± 1.10	98.95 ± 1.21	33.23 ± 1.35	67.83± 0.30
*C. loureiroi*	Infusion	35.63± 2.04	53.83 ± 1.51	59.47 ± 2.03	73.11 ± 1.61	33.65 ± 1.36	51.75 ± 0.99	74.52 ± 2.10	14.96 ± 2.50	44.41± 0.23
	Decoction	45.11± 1.14	54.81 ± 2.11	62.46 ± 0.49	82.34 ± 0.37	42.81 ± 6.33	63.95 ± 1.03	87.26 ± 2.85	27.23 ± 0.11	55.71± 1.17
	Ethanol extract	47.38± 2.72	67.53 ± 1.47	73.70 ± 1.89	99.99 ± 0.01	66.47 ± 0.09	80.46 ± 3.29	96.19 ± 1.37	32.24 ± 0.60	67.00± 0.64
Capsule from pharmacy		46.63± 1.50	59.17 ± 0.45	71.50 ± 0.39	98.68 ± 1.29	50.12 ± 0.07	78.36 ± 2.82	92.07 ± 1.26	28.31 ± 1.83	49.08± 0.72
Capsule from herbalist		37.46± 0.22	48.05 ± 0.40	71.03 ± 3.21	89.63 ± 3.58	27.78 ± 0.99	39.92 ± 3.21	73.45 ± 1.05	25.09 ± 1.69	47.83± 0.88
Bag tea		30.66± 4.58	36.38 ± 2.92	58.21 ± 1.92	78.18 ± 1.04	32.54 ± 2.09	69.75 ± 4.18	84.53 ± 1.43	17.89 ± 2.12	34.21 ± 2.50
Reference substance		77.99± 3.74(Acarbose)	93.73 ± 0.43 (Acarbose)	99.99 ± 0.01 (Acarbose)	99.99 ± 0.01 (Acarbose)	88.37 ± 1.72 (Acarbose)	96.15 ± 0.93 (Acarbose)	99.99 ± 0.01 (Acarbose)	87.46 ± 0.01 (Quercetin)	98.87 ± 0.10 (Quercetin)
